# Qualitative and Quantitative Estimation of Ceftriaxone in Pharmaceutical Dosage Forms Using Reverse Phase High Performance Liquid Chromatography

**DOI:** 10.31662/jmaj.2025-0309

**Published:** 2025-12-05

**Authors:** Hibba Dar, Rehana Tabassum, Younis Rather, Sameena Farhat

**Affiliations:** 1 Department of Pharmacology, Government Medical College, Srinagar, Jammu and Kashmir, India; 2 Multidisciplinary Research Unit, Government Medical College, Srinagar, Jammu and Kashmir, India

**Keywords:** ceftriaxone, RP-HPLC, method validation, drug quality assessment, substandard formulations

## Abstract

**Introduction:**

As the “pharmacy of the world,” India plays a critical role in global pharmaceutical supply. However, increasing reports of substandard and falsified medicines raise serious concerns. With Jammu and Kashmir witnessing repeated quality failures of ceftriaxone, this study aims to verify its quality using a refined high-performance liquid chromatography (HPLC)-based analytical method.

**Methods:**

The ceftriaxone assay was performed using Agilent 1260 Infinity HPLC System with a C18 column and EZChromS1 software. A solvent system of acetonitrile and water (20:80, v/v) was used, with flow rate of 0.5 mL/min and ultraviolet detection at 254 nm. Standard solutions (20-70 μg/mL) were prepared from a 1,000 μg/mL stock for calibration. Nineteen injectable ceftriaxone brands were procured and coded for anonymity. Sample solutions were prepared at 50 μg/mL. The method was validated as per International Council for Harmonisation (ICH) guidelines for accuracy, precision, linearity, specificity, limit of detection (LOD), limit of quantification (LOQ), and robustness. Solution stability was assessed over 7 days at 2°C-8°C.

**Results:**

The developed isocratic reversed-phase HPLC method showed good linearity (R^2^ = 0.9991) over the range 20-70 μg/mL, with LOD and LOQ of 5.88 μg/mL and 17.83 μg/mL, respectively. Intra-day and inter-day precision and accuracy showed percentage relative standard deviation (%RSD) values <2% and mean recovery within acceptable limits (96.9%-102.6%). Robustness was confirmed through variations in flow rate, mobile phase composition, detection wavelength, and column temperature, all yielding %RSD <2%. Analysis of 19 marketed formulations revealed that 89.5% complied with pharmacopoeial standards (90.0%-115.0% recovery), while 10.5% (CEF A17, CEF A19) were substandard, indicating significant quality variation among brands (p < 0.05).

**Conclusions:**

A simple, sensitive, and cost-effective HPLC method was developed and validated for estimation of ceftriaxone in pharmaceutical dosage forms. Among 19 tested formulations, 89.5% complied with Indian Pharmacopoeial standards, while two formulations were found substandard, indicating variability in product quality.

## Introduction

The aim of universal health coverage is that all people have access to the health services they need, when and where they need them, without financial hardship ^(1)^. To achieve this universal health coverage, the United Nations declared access to “safe, effective, quality, and affordable essential medicines and vaccines for all” as one of the Sustainable Development Goals (3.8) in their 2030 Agenda ^(2)^. Assuring the quality of medicines is crucial for providing safe and effective health care and reducing overall health care costs.

In terms of the pharmaceutical global market, India accounts for a significant share and has come to be known as the pharmacy of the world ^(3)^. By volume, India now ranks third worldwide when it comes to pharmaceutical production. The Indian pharmaceutical sector supplies over 50% of the global demand for various vaccines, 40% of the generic demand for the United States and 25% of all medicines for United Kingdom ^(4)^. Keeping in mind India’s growing position in the global pharmaceutical market, it becomes all the more imperative to keep a check on the quality of medicines being manufactured within the Indian pharmaceutical industry.

Molecules that are more commonly used, including antibiotics, have an increased likelihood of being counterfeited. In fact, among all classes of medicines, it was observed that quality issues in antimicrobials were reported 8-10 times more frequently ^(5)^. According to a World Health Organization (WHO) report released in 2017, 10.5% of medicines sold in low- and middle-income countries (LMICs), including India, are substandard and falsified ^(6)^. Beta-lactam antibiotics, especially penicillin and cephalosporins, have emerged as the most in-demand class of antimicrobials, with worldwide sales of approximately 65% of the total market of antibiotics ^(7)^. Beta-lactams also account for 50% of all reported cases of counterfeit antibiotics ^(8)^.

Among the substandard and falsified products reported to the WHO Global Surveillance and Monitoring System between 2013 and 2017, 17% are antibiotics ^(9)^. The prevalence of substandard antimicrobial agents varies from 0% to >50% globally ^(5)^. India has emerged as the top-most country in producing poor-quality antimicrobials ^(5), (8)^.

For assay and impurity profiling methods used in routine regulatory and quality control procedures, liquid chromatography―mainly high-performance liquid chromatography (HPLC) coupled with ultraviolet (UV) or fluorescence detection, has been established as the gold standard ^(10), (11), (12), (13)^.

In a study conducted by Shah et al. ^(14)^, 2013, a C18 column of 250 mm length and 4.6 mm diameter, with isocratic solvent delivery system was used. They employed a ternary mixture of acetonitrile (ACN), methanol, and triethyl amine buffer (pH 7) in ratio of 1:1:2 v/v as mobile phase, and analyzed the eluent at a wavelength of 240 nm, with a flow rate of 0.9 ml/min and a run time of 10 minutes. Gurupadayya and Disha ^(15)^, 2013, in their study, achieved chromatographic separation on a C8 column of 250 mm length, 4.6 mm diameter, and 5 μm particle size. The analyte was examined with a UV detector at 298 nm with a flow rate of 1 ml/min. The study conducted by Shah and Jat ^(16)^, 2015 used a mobile phase comprising buffer and methanol, pH 6.8 in the ratio of 90:10 (v/v). The eluent was analyzed at 260 nm, with a flow rate of 1 ml/min, and a run time of 20 minutes.

While these methods demonstrated acceptable validation parameters, they employed complex or buffer-based mobile phases, longer runtimes, and did not include real-world assessment of marketed formulations.

The ceftriaxone monograph in the Indian Pharmacopoeia specifies that a ceftriaxone injection contains not less than 90.0% and not more than 115.0% of the stated amount of ceftriaxone ^(17)^.

Looking at the increasing incidences of substandard drugs in the local market of Jammu and Kashmir, we have chosen ceftriaxone, which is used extensively at all levels of health care in the valley and the country, and has also been found to be “out of specification” limits on multiple occasions by the Drug and Food Control Organization of Jammu and Kashmir ^(18), (19), (20), (21), (22)^, to verify quantity and quality by employing reverse-phase HPLC (RP-HPLC)-based analytical technique.

Only a few analytical methods were reported for pharmaceutical preparations alone ^(15), (16), (23)^. Taking into consideration already reported methods, we aimed to improve upon validated methods for the estimation of ceftriaxone and develop a simple, precise, less time-consuming, and easily reproducible method which would have a wide linear range and good sensitivity for assay of ceftriaxone in the powder for injectable dosage forms.

## Materials and Methods

The HPLC 1260 Infinity Model from Agilent Technologies was used for analysis of pharmaceutical formulations. The column was analytical column C18 100/4.6 mm, 3.5 μm particle size, and 20 μL sample loop size. We used EZChromS1 software in our laboratory for HPLC data processing.

### Chemicals used

The reference sample ceftriaxone standard (pharmaceutical reference standard of 99.9% purity) was obtained from Sigma-Aldrich, Mumbai, India. ACN and HPLC-grade water was procured from Merck Specialties^Ⓡ^ Private Limited, Mumbai, India.

### Preparation of standard drug solution and plotting of calibration curve

Pure reference standard of ceftriaxone was used as the external standard in the analysis. Accurately weighed 50 mg of ceftriaxone was transferred to a 50 mL volumetric flask. Initially, 5 mL of the solvent system (ACN: Water; 20:80%, v/v) was added to the flask and placed on the vortex to ensure proper dissolution. This was then diluted up to the 50 mL mark to obtain a final concentration of ceftriaxone standard of 1,000 μg/mL as standard stock solution.

Standard calibration solutions were prepared from the stock solution (20 μg/mL, 30 μg/mL, 40 μg/mL, 50 μg/mL, 60 μg/mL, and 70 μg/mL) and a 6-point calibration curve was constructed.

### Preparation of sample solution

The injectable formulation of ceftriaxone from 19 different brands was procured in triplicates from pharmacies located in urban and rural areas of different districts of Kashmir, India. Brands of ceftriaxone included in our study were numerically coded to obscure name and batch number to maintain anonymity of the brands (code number of brands tested: CEF A1-CEF A19). Stock solutions for the ceftriaxone formulations were prepared having concentration of 1,000 μg/mL. From the stock solutions, a dilution of 50 μg/mL was prepared in triplicate for estimation.

### Preparation of solvent system (pre-mixing)

The isocratic solvent system was prepared by adding 200 mL of HPLC-grade ACN to 800 mL of HPLC-grade water in a 1,000 mL volumetric flask in a ratio of 20:80% v/v.

### Method development and validation

Initially, various mobile phase compositions of ACN and water with 1% acetic acid (ratios of 20:80, 25:75, and 30:70) were tested, but they resulted in undesirable changes in peak symmetry, finning, and retention time.

The RP-HPLC method for ceftriaxone assay was validated in terms of accuracy, precision, reproducibility, linearity, specificity, limit of detection (LOD), limit of quantification (LOQ), and robustness according to ICH Harmonized Tripartite Guidelines. To evaluate accuracy of the developed method, successive analysis (n = 3) of three different concentrations (20 μg/mL, 40 μg/mL, and 60 μg/mL) of standard ceftriaxone solution were performed at different times during the same day and on different days. Precision was evaluated by analyzing the standard drug at three concentration levels (20 μg/mL, 40 μg/mL, and 60 μg/mL). Each concentration was analyzed in triplicate at different times during the same day (intra-day precision - repeatability) and on different days (inter-day precision - intermediate precision). Percent recovery, standard deviation (SD), and relative standard deviations (RSDs) were calculated. Standard curves were constructed at concentrations 20, 30, 40, 50, 60, and 70 μg/mL of ceftriaxone.

### Study of solution stability

The stability of the analytical solutions was determined by analyzing the ceftriaxone standard solution immediately after preparation. The prepared solution was stored at a temperature of 2°C-8°C and analyzed 7 days apart. Percentage (%) RSD values were calculated using a specification limit of ±2% from the initial value.

## Results

### Development of chromatographic system

The liquid chromatographic method was isocratic in nature, employing a mobile phase mixture of ACN and water in a ratio of 20:80% v/v. The flow rate of our mobile phase was set at 0.5 mL/min and UV detection was monitored at a wavelength of 254 nm. The injection volume was 20 μL with a run time of 5 minutes.

### Method validation

Validation of the optimized method was performed in accordance with ICH Q2 (R1) guidelines. The validation characteristics that were addressed included specificity, LOD, LOQ, linearity and range, precision and accuracy, robustness, and solution stability.

### Specificity

**Figure 1.**
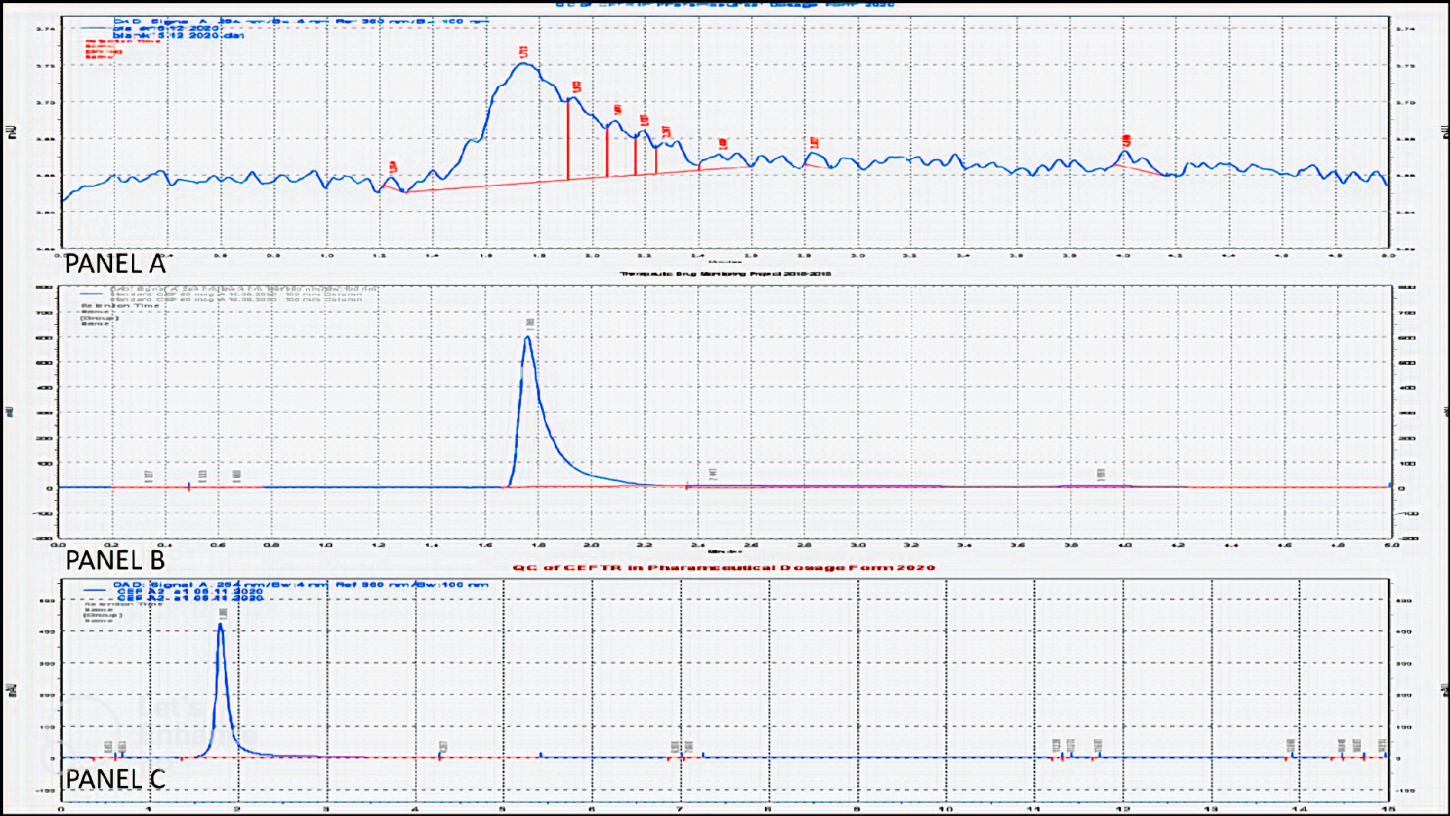
Panel A - Representative blank chromatogram (run time 5 minutes). (image of blank chromatogram has been magnified to show values of milli absorbance units closer to zero). Panel B - Representative chromatogram of ceftriaxone standard at concentration of 50 μg/mL (run time 5 minutes). Panel C - Representative chromatogram of ceftriaxone in dosage formulation at concentration of 50 μg/mL (run time 15 minutes).

Specificity of the RP-HPLC method was assessed by checking that no interference peaks were found at the retention time of ceftriaxone with mobile phase blank, standard and sample solutions. For this, chromatograms of solutions of standard (50 μg/mL), sample (50 μg/mL), and mobile phase blanks were compared (Figure 1).

### LOD and LOQ

LOD is the lowest concentration in a sample that can be detected, but not necessarily quantified under the stated experimental conditions. LOQ is the lowest concentration of analyte that can be determined with acceptable precision and accuracy. The LOD (3.3 SD/S) and LOQ (10 SD/S) were then calculated on the basis of SD of response and slope (S) of the calibration curve. An LOD value of 5.88 μg/mL was obtained. The LOQ value in our developed method was 17.83 μg/mL.

### Linearity and range

Standard curves were constructed at concentrations 20, 30, 40, 50, 60, and 70 μg/mL of ceftriaxone. A linear correlation was found between absorbance at λmax (254 nm) and concentrations of ceftriaxone in the range of 20-70 μg/mL. Curves were obtained by plotting the peak area against concentrations of ceftriaxone. Linear calibration curves were generated by linear regression analysis and obtained over the respective standard concentration ranges. The suitability of the calibration models was confirmed by back-calculating the concentrations of the calibration standards. The regression statistics are shown in Table 1.

**Table 1.** Linearity Data of Developed Method.

Parameters	Values
λ max (nm)	254
Linearity range (μg/ml)	20-70
Percent recovery	100.1
Standard deviation of % recovery	1.71
%RSD	1.7
LOD (μg/mL)	5.88
LOQ (μg/mL)	17.83
Slope	212,948
Standard error on slope	3,222.16
Confidence limit of slope (95%)	204,002.25-221,894.55
Intercept	−1,372,394 (−1E + 06)
Standard error on intercept	155,088.27
Confidence limit of intercept (95%)	−1,802,988.36 to −941,800.22
Coefficient of determination (R^2^)	0.9991

Λ max: detection wavelength; %RSD: percent relative standard deviation; LOD: limit of detection; LOQ: limit of quantitation. Table 1 contains the linearity data of our developed method obtained by applying linear regression statistics over the concentration range of 20-70 μg/mL.

### Precision and accuracy

The precision of the developed method was evaluated by analyzing the pure standard drug at three different concentration levels within the linear range (20 μg/mL, 40 μg/mL, and 60 μg/mL). Each concentration was analyzed in triplicates at different times during the same day (intra-day precision - repeatability) and on different days (inter-day precision - intermediate precision). Percent recovery, SD, and RSDs were calculated. The results obtained were tabulated along with standard error and 95% confidence interval. Precision data for intra-day assay (repeatability) showed %RSD of 1.59%, while data for inter-day assay (intermediate precision) showed %RSD of 1.16%. (Table 2)

**Table 2.** Validation Parameters of Ceftriaxone by RP-HPLC.

Parameters		RP-HPLC method	Recommended limits	
Specificity		Chromatograms enclosed	No interferences	
Accuracy, %		99.4%	95.0%-105.0%	
Precision	Intra-day Inter-day	1.59 1.16	NMT 2.00 %RSD	
Robustness	Flow rate (mL/min)	0.4 0.6	0.96 0.48	NMT 2.00 %RSD
	%ACN	15 25	0.39 1.00	
	Wavelength (nm)	250 258	0.06 0.40	
	Column temperature	30°C 40°C	0.87 0.40	

**Figure 2.**
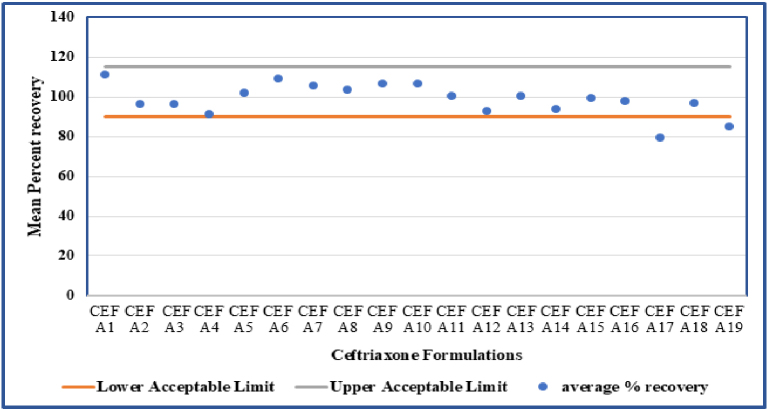
Shows average percent recovery of the ceftriaxone formulations. The two target lines indicate permissible limits of ceftriaxone as according to the Indian Pharmacopoeia (90%-115%). It is clear that average percent recovery of formulations CEF A17 and CEF A19 fall below the lower permissible limit.

%RSD: percent relative standard deviation; NMT: not more than. Table 2 depicts the parameters evaluated in the validation of our developed method. For specificity of the method, relevant chromatograms have been enclosed (Figure 2). Mean accuracy of the method is 99.45% and %RSD for precision studies and robustness have values <2.00.

Accuracy is obtained by calculating the percent recovery of the analyte recovered. To evaluate the accuracy of the developed method, successive analysis (n = 3) of three different concentrations (20 μg/mL, 40 μg/mL, and 60 μg/mL), within the linear range of standard ceftriaxone solution was performed at different times during the same day and on different days. The data of the experiment was statistically analyzed to study the recovery and validity of the developed method. The mean recovery should be within 90.0%-115.0% to be accepted (Table 2).

### Robustness

The robustness of the method was studied by deliberate changes in the method like alterations in flow rate (0.4, 0.5, 0.6 mL/min), alteration in percent of ACN in the mobile phase (15%, 20%, 25%), changes in the wavelength (250, 254, 258 nm), and changes in column temperature (30°C, 35°C, 40°C). Representative chromatograms obtained from the robustness studies have been provided in the Supplementary Material. Data acquired from these minor variations showed %RSD values of <2% in all parameters.

### Solution stability

The stability of the analytical solutions was determined by analyzing the ceftriaxone standard solution, stored at a temperature of 2°C-8°C 7 days apart. %RSD values of the results were calculated with the specification limit that final assay value should be within 2% of the initial value.

A consolidated summary of the validation results is presented in Table 2, while detailed data has been provided in the Supplementary Material. As shown in Table 2, the method exhibited good specificity with no interference from excipients, and the mean accuracy was found to be 99.4%, which is within the acceptable range of 95.0%-105.0%. Precision, both intra-day and inter-day, demonstrated %RSD values <2.00, indicating the reproducibility of the method. The method also proved robust under deliberate variations in flow rate, mobile phase composition, detection wavelength, and column temperature, with all %RSD values remaining well below the 2.00 threshold.

These results confirm that the developed method is reliable and suitable for routine quantitative analysis of ceftriaxone in pharmaceutical formulations.

### Analysis of pharmaceutical formulations

For quantitative and qualitative analysis of ceftriaxone in pharmaceutical dosage form, we injected equal volumes of the standard and test preparation, recorded the chromatograms, and evaluated the responses for the peak of interest. Out of the 19 ceftriaxone formulations, 89.5% of the formulations (n = 17) were within the range of the Indian Pharmacopoeia specification, that is 90.0%-115.0%, while 10.5% (n = 2) of the formulations were found to be out of specification limits (Figure 2).

**Figure 3.**
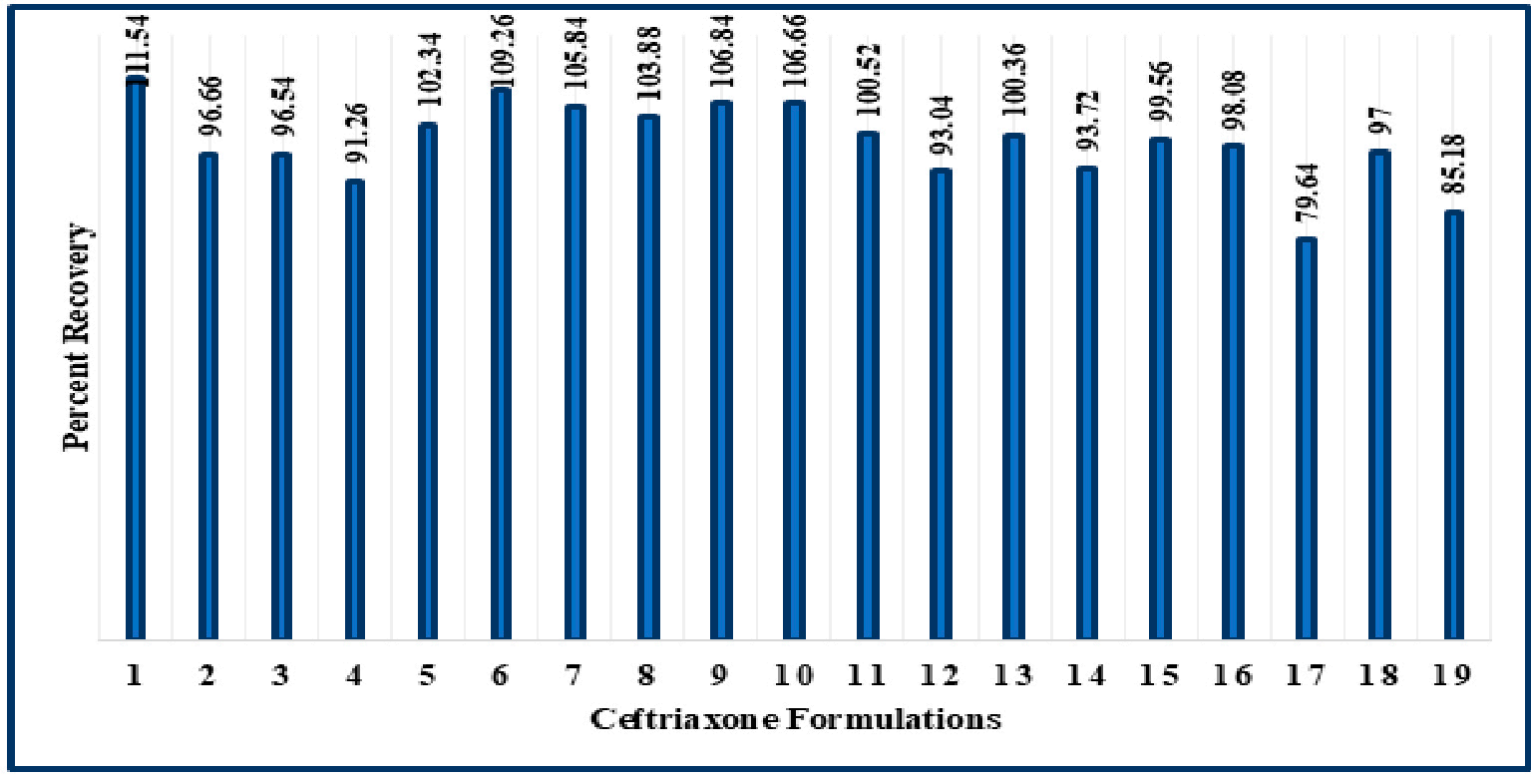
Depiction of average percent recovery of ceftriaxone formulations CEF A1-CEF A19.

**Figure 4.**
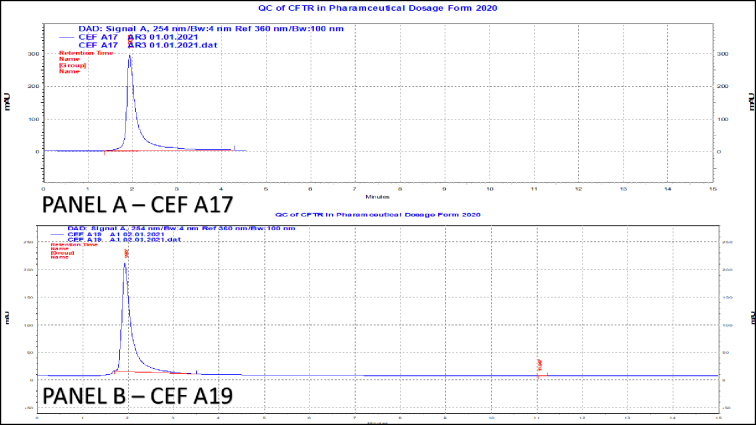
Panel A - Representative chromatogram of substandard formulation-CEF A17. Panel B - Representative chromatogram of substandard formulation-CEF A19.

**Figure 5.**
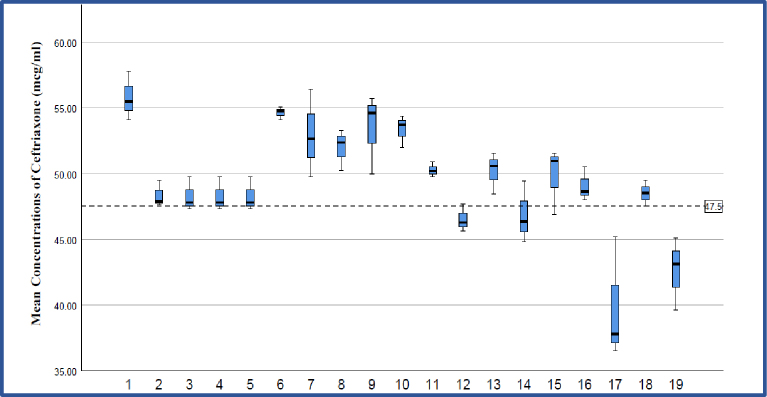
Shows a simple boxplot of mean obtained concentrations of ceftriaxone in different formulations at expected concentration of 50 μg/mL. It is clear from the box plot that degree of dispersion (spread) is at the minimum in CEF A6 and CEF A11, and at the maximum in CEF A17.

At expected concentration of 50 μg/ml, all the formulations contained ceftriaxone as per the standards of the Indian Pharmacopoeia (90.0%-115.0%), except for two formulations, CEF A17 and CEF A19. CEF A17 (mean percent recovery 79.6%) and CEF A19 (mean percent recovery 85.1%) were found to contain ceftriaxone below the lower permissible limit of ceftriaxone (Figure 3 and 4). At an expected concentration of 50 μg/mL of ceftriaxone, p value is statistically significant (p < 0.05). This indicates that there is statistically significant difference between the obtained mean concentrations of ceftriaxone in different formulations (Table 3). At an expected concentration of 50 μg/mL, the degree of dispersion (spread) is at the minimum in CEF A6 and CEF A11, and at the maximum in CEF A17 (Figure 5).

**Table 3.** Multiple Comparisons - Bonferroni.

Dependent variable: mean							
Expected concentration (CEF)			Mean difference (I-J)	Std. error	Sig.	95% Confidence interval	
						Lower bound	Upper bound
50	Ceftriaxone standard	CEF A1	−5.54667	1.66941	0.364	−12.2301	1.1367
		CEF A2	1.89000	1.66941	1.000	−4.7934	8.5734
		CEF A3	1.94667	1.66941	1.000	−4.7367	8.6301
		CEF A4	1.94667	1.66941	1.000	−4.7367	8.6301
		CEF A5	1.94667	1.66941	1.000	−4.7367	8.6301
		CEF A6	−4.41333	1.66941	1.000	−11.0967	2.2701
		CEF A7	−2.70333	1.66941	1.000	−9.3867	3.9801
		CEF A8	−1.72333	1.66941	1.000	−8.4067	4.9601
		CEF A9	−3.19667	1.66941	1.000	−9.8801	3.4867
		CEF A10	−3.11333	1.66941	1.000	−9.7967	3.5701
		CEF A11	−0.04333	1.66941	1.000	−6.7267	6.6401
		CEF A12	3.69667	1.66941	1.000	−2.9867	10.3801
		CEF A13	0.04000	1.66941	1.000	−6.6434	6.7234
		CEF A14	3.36000	1.66941	1.000	−3.3234	10.0434
		CEF A15	0.44000	1.66941	1.000	−6.2434	7.1234
		CEF A16	11.17667	1.66941	1.000	−5.5067	7.8601
		CEF A17	10.39667*	1.66941	0.000	3.7133	17.0801
		CEF A18	1.71667	1.66941	1.000	−4.9667	8.4001
		CEF A19	7.62667*	1.66941	0.009	0.9433	14.3101

*The mean difference is significant at the 0.05 level. In Table 3, it is clear that by comparing the ceftriaxone standard with all 19 ceftriaxone formulations at expected concentration of 50 μg/mL of ceftriaxone, there is a statistically insignificant mean difference between the different formulations, except in case of following, where the mean difference is statistically significant; CEF standard and CEF A17 (p < 0.05), CEF standard and CEF A19 (p < 0.05).

## Discussion

The developed RP-HPLC method for ceftriaxone was optimized using a relatively straightforward ACN-water system without the need for buffer or additional modifiers. This enabled us to achieve a simplified preparation, which would significantly improve reproducibility. Further, this study bridges the gap between analytical validation and public health by applying a validated method to assess market samples, revealing compliance failures in real-world settings.

One of the key challenges encountered during method development was the optimization of the mobile phase. Initially, various compositions of ACN and water with 1% acetic acid were evaluated in ratios of 20:80, 25:75, and 30:70. However, these combinations led to undesirable chromatographic behaviors such as poor peak symmetry, finning, broadened peaks, and inconsistent retention times. These anomalies significantly hindered the reproducibility and clarity of the chromatograms, making them unsuitable for reliable quantification. Ultimately, the removal of acetic acid from the mobile phase and adoption of a simpler ACN:water system in a 20:80 ratio resulted in well-resolved, symmetric peaks with stable retention times and improved reproducibility―thus forming the basis of the finalized isocratic method. Our validated method provided comparable accuracy and precision, while working at a shorter runtime (5 minutes), had a simpler mobile phase, with robustness and stability within acceptable limits.

The validation results confirmed that the method is specific, sensitive (LOD = 5.88 μg/mL), accurate (99.4% mean recovery), precise (%RSD < 2%), robust, and stable under refrigerated storage.

The results of analysis of commercial formulations agrees with findings observed in a WHO report released in 2017, wherein 10.5% of medicines sold in LMIC countries, including India, were found to be substandard and falsified ^(6)^. Similar findings were also published in a report released by the Ministry of Health and Family Welfare, Government of India in 2003, wherein it was estimated that counterfeit drugs account for 0.34% of the total pharmaceutical market and substandard drugs account for 9.34% (results varied from 8.19% to 10.64%) of the drug market in India ^(24)^.

In a study conducted by Ali, 2009 ^(25)^, out of 96 samples of ceftriaxone (of strengths 250, 500, and 1,000 mg), belonging to 33 brands, 15.62% samples (n = 15) were found out of specification range (i.e., 90.0%-115.0%). A study conducted by Sotade et al. ^(26)^, 2017 estimated an average percent recovery of 13 formulations of ceftriaxone to be between 100.3% and 103.8%. From their assay, it was concluded that the content of active ingredients was within the specified pharmacopoeial range. In a study conducted by Shah and Jat ^(16)^, 2015, the estimated average recovery percent of ceftriaxone was between 99.4% and 99.6% (n = 3). Shah et al. ^(14)^, 2013, in their study, obtained an average recovery percent of ceftriaxone of between 96.9% and 102.1% (n = 2). Arnet et al. ^(27)^, 2014 tested eight generic ceftriaxone products manufactured in Eastern Asia and found that all eight preparations failed to meet in vitro quality standards when compared to the original branded product.

Despite concerns regarding the perceived routine nature of the work, the novelty of our study lies in the application of a validated analytical method to real-world market surveillance in a region (Jammu and Kashmir) known for recurrent quality issues in antibiotics. By analyzing 19 marketed brands and identifying two as substandard, our work underscores the public health importance of simple, robust, and reproducible quality control tools. Unlike purely analytical studies focused on method development, our study integrates method validation with post-marketing surveillance, providing regulatory insights and demonstrating continued risk of substandard formulations in the pharmaceutical supply chain.

The detection of substandard products (CEF A17 and CEF A19) underscores the need for stringent quality control and post-marketing surveillance to ensure patient safety.

### Limitations

While the method showed acceptable validation characteristics, it was optimized for ceftriaxone alone and not designed for detection of degradation products or related impurities. The study was limited to a single geographical area (Jammu and Kashmir), and broader market surveillance may be required to generalize the findings. Matrix effects from excipients in complex formulations were not explored.

### Conclusion

A robust, cost-effective RP-HPLC method for ceftriaxone estimation in pharmaceutical dosage form was developed and validated as per ICH guidelines. The method showed high specificity, precision, and robustness. Application to 19 brands of ceftriaxone revealed that 10.5% failed to meet Indian Pharmacopoeial standards, highlighting the need for improved regulatory oversight and routine quality control.

## Article Information

### Author Contributions

Younis Rather contributed to the conception and design of this study. Younis Rather and Hibba Dar performed the analysis of the pharmaceutical formulations. Hibba Dar did the statistical analyses and data management, and drafted the manuscript. Sameena Farhat and Rehana Tabassum contributed to the concept of this study and gave advice on the interpretation of the study results. Younis Rather and Rehana Tabassum reviewed the manuscript and supervised the whole study process. All authors read and approved the final manuscript.

### Conflicts of Interest

None

### Approval by Institutional Review Board (IRB)

This study was approved by the Institutional Ethical Committee of Government Medical College, Srinagar with Approval code IRBGMC-SGR/Pharma/913.

## Supplement

Supplementary Material
