# Supplementary material for: Qualitative and Quantitative Estimation of Ceftriaxone in Pharmaceutical Dosage Forms Using Reverse Phase High Performance Liquid Chromatography
Source: JMA J. 2025 Dec 5;9(1):292–301. doi: 10.31662/jmaj.2025-0309 (PMC12889181; doi:10.31662/jmaj.2025-0309)
Supplement: Supplementary Material [file 2433-3298-9-1-0292-s001.pdf]

SUPPLEMENTARY MATERIAL

Table 4: Analytical parameters for determination of ceftriaxone	
**System Parameters**	**Values**
**Stationary Phase**	Octadecylsilane (C18)
**Column dimensions**	100 mm x 4.6 mm, 3.5 µm particle size
**Elution**	Isocratic (Acetonitrile: Water (20:80%, v/v)
**Injection volume**	20 µL
**Flow rate**	0.5 ml/min
**Detection Wavelength (λ max)**	254 nm
**Column Temperature**	35°C
**Run time**	5 minutes
**Concentration of test solution**	50 µg /ml

Table 4 enlists the chromatographic settings of our developed method.

Table 5: Inter-day and intraday precision study data								
**Drug**	**Type of Precision**	**Conc. Taken (µg/mL)**	**Conc. obtained (µg/mL)**	**SD**	**% RSD**	**SE**	**95% CI**	
							**Lower CI**	**Upper CI**
**Ceftriaxone**	**Intra day**	20	20.53	0.38	1.87	0.2222	20.15	20.92
		40	38.79	0.62	1.62	0.3633	38.16	39.41
		60	59.22	0.76	1.29	0.4437	58.45	59.98
	**Inter day**	20	20.18	0.34	1.68	0.1963	19.84	20.52
		40	38.78	0.19	0.49	0.1099	38.59	38.97
		60	59.36	0.78	1.32	0.4536	58.58	60.15

Each result is the average of separate triplicate analysis; (n = 3); SD: standard deviation; % RSD: percentage relative standard deviation; S.E: standard error; 95% CI: 95 percent confidence interval.
Table 5 shows the data obtained during interday and intraday precision tests performed for validation of our developed method.

Table 6: Inter-day and Intraday Accuracy study data				
**Drug**	**Type of Accuracy**	**Conc. Taken (µg /ml)**	**Conc. Obtained (µg /ml)**	**% Recovery**
**Ceftriaxone**	Intra day	20	20.53	102.68
		40	38.79	96.97
		60	59.22	98.70
	Inter day	20	20.18	100.9
		40	38.78	96.95
		60	59.36	98.93

Each result is the average of separate triplicate analysis; (n = 3);
Table 6 depicts percent recovery of ceftriaxone standard obtained while performing accuracy tests of our developed method.

Table 7(a): Data for Robustness of the Method – Parameter 1				
**Parameter**		**RT (mins)**	**Mean % recovery**	**% RSD**
**Flow Rate (ml/min)**	**0.4**	1.83	99.4	0.962
	**0.5**	1.8	100.1	0.238
	**0.6**	1.78	99.3	0.480

Table 7(a) depicts data obtained as part of robustness studies performed for validation of our developed method, wherein flow rate was altered (0.4 and 0.6 ml/min).

Table 7(b): Data for Robustness of the Method – Parameter 2				
**Parameter**		**RT (mins)**	**Mean % recovery**	**% RSD**
**% ACN in mobile phase**	**15**	1.83	95.7	0.396
	**20**	1.8	100.1	0.238
	**25**	1.76	96.4	1.001

Table 7(b) shows data obtained from robustness studies performed for validation of our developed method, wherein mobile phase composition was slightly altered (15 and 25 %).

Table 7(c): Data for Robustness of the Method – Parameter 3				
**Parameter**		**RT (mins)**	**Mean % recovery**	**% RSD**
**Wavelength (nm)**	**250 nm**	1.78	96	0.068
	**254 nm**	1.8	100.1	0.238
	**258 nm**	1.8	95.6	0.405

Table 7(c) indicates data obtained as part of the robustness study, where the wavelength for detection was varied (250 and 258 nm).

Table 7(d): Data for Robustness of the Method – Parameter 4

Parameter		RT (mins)	Mean % recovery	% RSD
Column Temperature	**30°C**	1.77	96.7	0.874
	**35°C**	1.8	100.1	0.238
	**40°C**	1.78	95.9	0.407

Table 7(d) depicts data obtained from robustness studies for validation of our developed method, where slight variations were made in column temperature (30°C and 40°C).

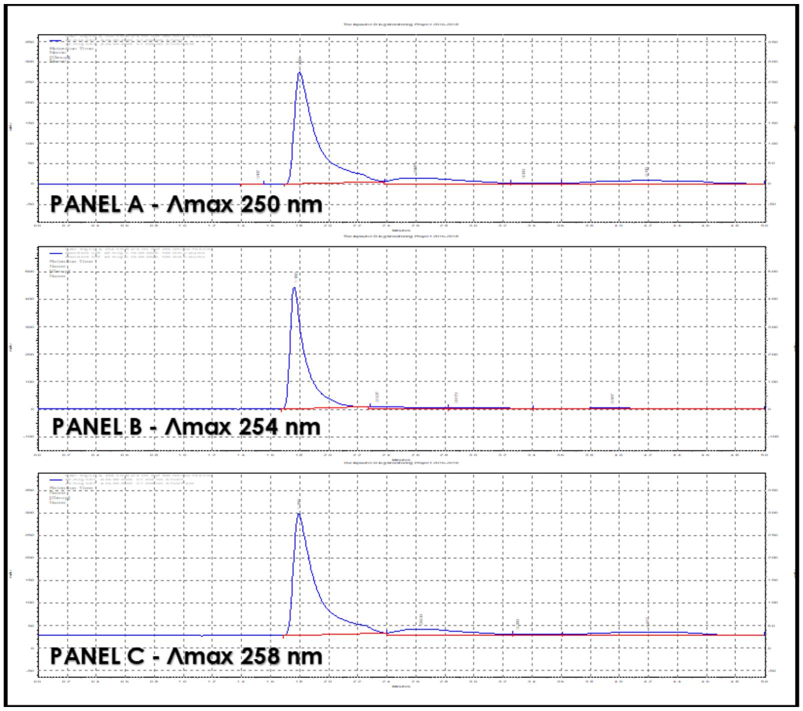

Figure 6: Representative chromatograms of ceftriaxone standard at concentration of 40 µg /ml, obtained while studying robustness of the developed method by deliberate changes in wavelength.

Panel A - Representative chromatogram of ceftriaxone standard at concentration of 40 µg /ml, with λmax of 250nm.

Panel B - Representative chromatogram of ceftriaxone standard at concentration of 40 µg /ml, with λmax of 254nm.

Panel C - Representative chromatogram of ceftriaxone standard at concentration of 40 µg /ml, with λmax of 258nm.

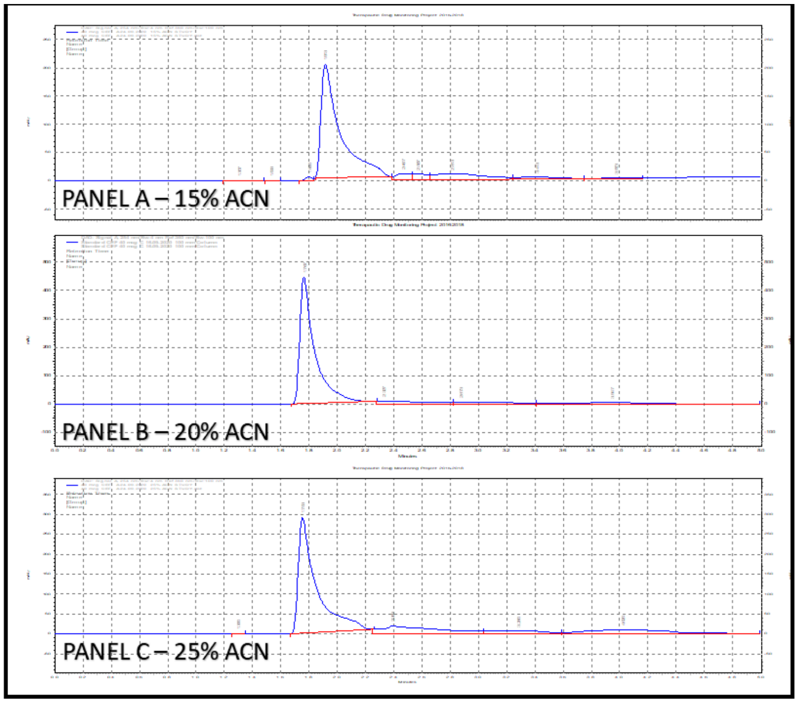

Figure 7: Representative chromatograms of ceftriaxone standard at concentration of 40 µg /ml, obtained while studying robustness of the developed method by alteration in percent of ACN in the mobile phase.

Panel A - Representative chromatogram of ceftriaxone standard at concentration of 40 µg /ml, with 15% ACN.

Panel B - Representative chromatogram of ceftriaxone standard at concentration of 40 µg /ml, with 20% ACN.

Panel C - Representative chromatogram of ceftriaxone standard at concentration of 40 µg /ml, with 25% ACN.

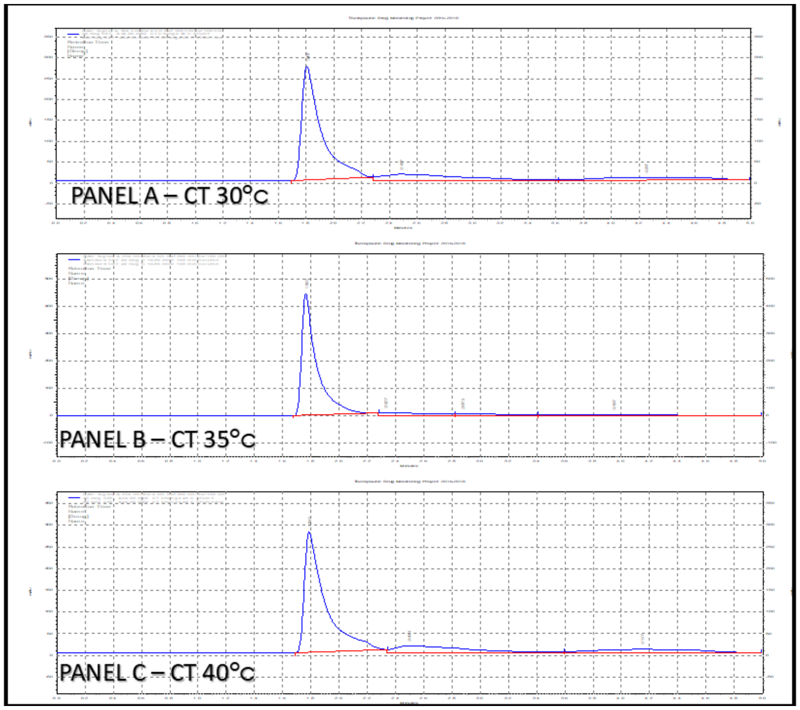

Figure 8: Representative chromatograms of ceftriaxone standard at concentration of 40 µg /ml, obtained while studying robustness of the developed method by changes in column temperature.

Panel A - Representative chromatogram of ceftriaxone standard at concentration of 40 µg /ml, with column temperature of 30℃.

Panel B - Representative chromatogram of ceftriaxone standard at concentration of 40 µg /ml, with column temperature of 35℃.

Panel C - Representative chromatogram of ceftriaxone standard at concentration of 40 µg /ml, with column temperature of 40℃.

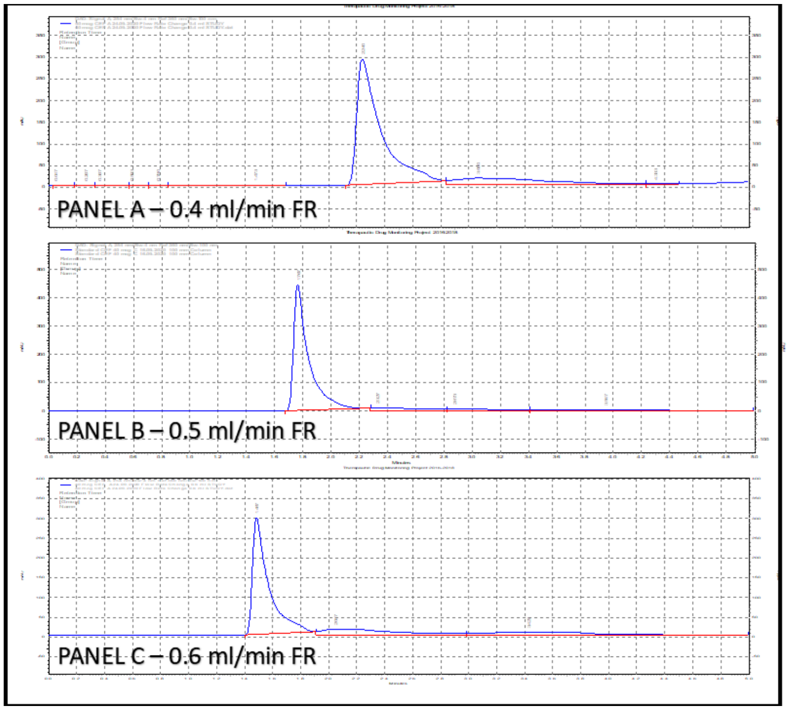

Figure 9: Representative chromatograms of ceftriaxone standard at concentration of 40 µg /ml, obtained while studying robustness of the developed method by alterations in flow rate.

Panel A - Representative chromatogram of ceftriaxone standard at concentration of 40 µg /ml, with flow rate of 0.4 mL/min.

Panel B - Representative chromatogram of ceftriaxone standard at concentration of 40 µg /ml, with flow rate of 0.5 mL/min.

Panel C - Representative chromatogram of ceftriaxone standard at concentration of 40 µg /ml, with flow rate of 0.6 mL/min.
